# Supplementary material for: Influence of NAFLD and bariatric surgery on hepatic and adipose tissue mitochondrial biogenesis and respiration
Source: Nat Commun. 2022 May 25;13:2931. doi: 10.1038/s41467-022-30629-5 (PMC9132900; doi:10.1038/s41467-022-30629-5)

Supplementary Information

Methods

Study participants

Guidelines for bariatric surgery in Denmark: Prior to 1st July 2017, criteria set by the Danish National Board of Health for RYGB or SG surgery were as follows: Age > 25 years and BMI > 35 kg/m^2, plus at least one of the following obesity-related complications: documented type-2 diabetes, hypertension, documented sleep apnea requiring nightly Continuous Positive Airway Pressure (CPAP), documented polycystic ovarian syndrome, documented arthrosis in lower extremities *or* age > 25 years and a BMI > 50 kg/m^2 without obesity-related comorbidities.

After 1st July 2017, new guidelines for referral for bariatric surgery were published. The current criteria are as follows: Age > 18 and BMI > 35 kg/m^2, plus obesity-related comorbidities as listed above *or* a BMI > 40 kg/m^2 without obesity-related complications. Individual assessment is especially recommended for patients 18-25 years old.

Study-specific exclusion criteria were: current or previous alcohol consumption of > 2.5 units/day for men and > 1.5 units/day for women, preexisting liver disease other than NAFLD, preexisting disease in the lipid metabolism and acute or chronic inflammatory disease, or an ethnic origin other than North European.

Biochemical analyses

Plasma alanine aminotransferase (ALT), aspartate aminotransferase (AST), plasma-glucose and triglycerides were measured using the Roche/Hitachi Cobas c 8000 system (Roche Diagnostics GmbH, Mannheim, Germany) with Cobas calibrators and reagents, according to the manufacturer’s instructions. Serum insulin and c-peptide concentrations were measured by immunoassay Cobas e 602. HbA1c was measured in plasma with the Tosoh TSKgel G8 Variant His on the Tosoh Automated Glycohemoglobin Analyzer HLC-723G8 (Tosoh Corporation, Tokyo, Japan). All markers were measured in the fasting state.

Plasma inflammatory markers

Adiponectin (Cat. No. DRP300, R&D Systems, McKinley Place NE, Minneapolis), leptin (Cat. No. DLP00, R&D Systems, McKinley Place NE, Minneapolis), sCD163 (Cat. No. DC1630, R&D Systems, McKinley Place NE, Minneapolis) and sCD206 (Cat. No. DLP00, R&D Systems, McKinley Place NE, Minneapolis) were measured by sandwich ELISA (Cat. No. ELH-MMR, RayBiotech, Norcross, Georgia). Leptin were measured by a sandwich ELISA (Cat. No. DLP00, R&D Systems, McKinley Place NE, Minneapolis)

IL-1β, IL-6, and TNF-α were measured by customized electrochemiluminescence (ECL) assay (Cat. No. K151A9H-1, Mesoscale (MSD), Rockville, Maryland). sCD163 was measured in Hep-Plasma and the remaining inflammatory markers in cooled EDTA plasma. All markers were measured in the fasting state.

Quantification of mitochondrial DNA

To isolate DNA, 5-20mg of adipose or liver tissue was disrupted in 250 ul of alkaline lysis solution (25 mM NaOH and 2 mM EDTA) using a QIAGEN TissueLyser II bead homogenizer with two consecutive one-minute rounds at 30 hertz. After homogenization, samples were heated for one

hour at 96°C, and then neutralized with 250 ul of neutralization buffer (40 mM Tris-HCl). After centrifugation at 12,000 x g, the supernatant was carefully transferred to a new tube.

MtDNA primer sequence

mtCO2 F: Tgaagcccccattcgtataa

mtCO2 R: Cgggaattgcatctgttttt

mtCYTB F: Agacagtcccaccctcacac

mtCYTB R: Ggtgattcctagggggttgt

nUCP1 F: Gcccaatgaatactgccact

nUCP1 R: Tgcatgcattctaggtctttaattt

nPPARG F: Ttcagaaatgccttgcagtg

nPPARG R: Acatttttggcaatggcttt

Supplementary Table 1: Overview of HRR SUIT P1 and P2 measurements

**SUIT P1: Liver, VAT and SAT**
**GM step.** Leak respiration measured after addition of glutamate and malate; electron flow through *complex 1* as a measure of oxygen consumption used for compensating the electron leak through the inner mitochondrial membrane.
**GM_D step** Addition of ADP to initiate the oxidative respiratory process.
**GMO_D step**. State 3 *complex II* lipid respiratory capacity was measured after further addition of the fatty acid octanoyl carnitine.
**$GMOS_D$ ($OXPHOS_{max}$)**: Addition of succinate to achieve maximal mitochondrial coupled oxidative phosphorylation through complex I and II.

**FCCP step**. Testing of uncoupling capacity, hence a measure of maximal mitochondrial electron transport chain system capacity (ETS).
**SUIT P2 (only liver tissue)**
**GM step.** As in P1
**GM_D step.** As in P1
**Rotenone step**. Blocking of electron flow from complex I. Flux rates yielded here represent complex I leak state.
**Succinate step.** Donation of electrons to complex II. With complex I blocked with rotenone this step represents specific complex II capacity.
**Antimycin A step.** Blocking of electron flow from complex III. Flux rates yielded here represent complex III leak state.
**TMPD ascorbic acid step.** Powerful stimulator of complex IV. With blocking of complex III by Antimycin A in the previous step, specific complex IV capacity is yielded in this step.

Results

Statistical analyses

Upon request from one of the reviewers we have performed post hoc analyses of DM2 status, age and sex in the multiple lineary regression analyses alongside the chosen 4 predictors for each of the three tissues: liver, VAT and SAT.

Dependent variable: $OXPHOS_{max}$ per mtDNA: Multiple linear regression with forward selection.

Liver tissue:

Independent variables: BMI, leptin, ALT, liver steatosis grade, age, sex and DM2 status:

Adjusted R^2: 0.15, P=0.002.

BMI only significant predictor: Unstandardized β = 0.0012, 95% CI = 0.0005-0.0020, P=0.002

Excluded variables: Leptin, ALT, liver steatosis grade, age, sex and DM2 status:

VAT:

Independent variables: BMI, sCD163, adiponectin, HOMA-IR, age, sex and DM2 status:

Adjusted R^2: 0.54, P=0.050

sCD163 was the variable with the lowest p-value (P=0.050): Unstandardized β = $-4.39*10^{-6}$, 95% CI -0.000004 – $-2.3*10^{-9}$.

SAT:

Independent variables: BMI, sCD163, adiponectin, Hba1C, age, sex, DM2 status:

Adjusted R^2: 0.091, P-value 0.025.

BMI was the variable with the lowest p-value (P=0.025): Unstandardized β = -0.000063, 95% CI -0.00118 – -0.000009.

Dependent variable	Independent variable	Unstandardized β (95% CI)	Adjusted R^2	P-value
$OXPHOS_{max}$ per mtDNA	Type of surgery SG = 0 RYGB =1	-0.015 (-0.077-0.047)	-0.044	0.626
$OXPHOS_{max}$ per mtDNA	Delta NAS	-0.0001 (-0.027-0.027)	-0.059	0.993
$OXPHOS_{max}$ per mtDNA	Total weight loss	-0.002 (-0.004-0.0004)	0.098	0.104
Pure Complex II (Succinate P2)	Type of surgery SG = 0 RYGB =1	-0.045 (-0.148-0.058)	-0.001	0.347
Pure Complex II (Succinate P2)	Delta NAS	0.012 (-0.035-0.058)	-0.074	0.590

Supplementary figures

Supplementary Figure S1: Pig data

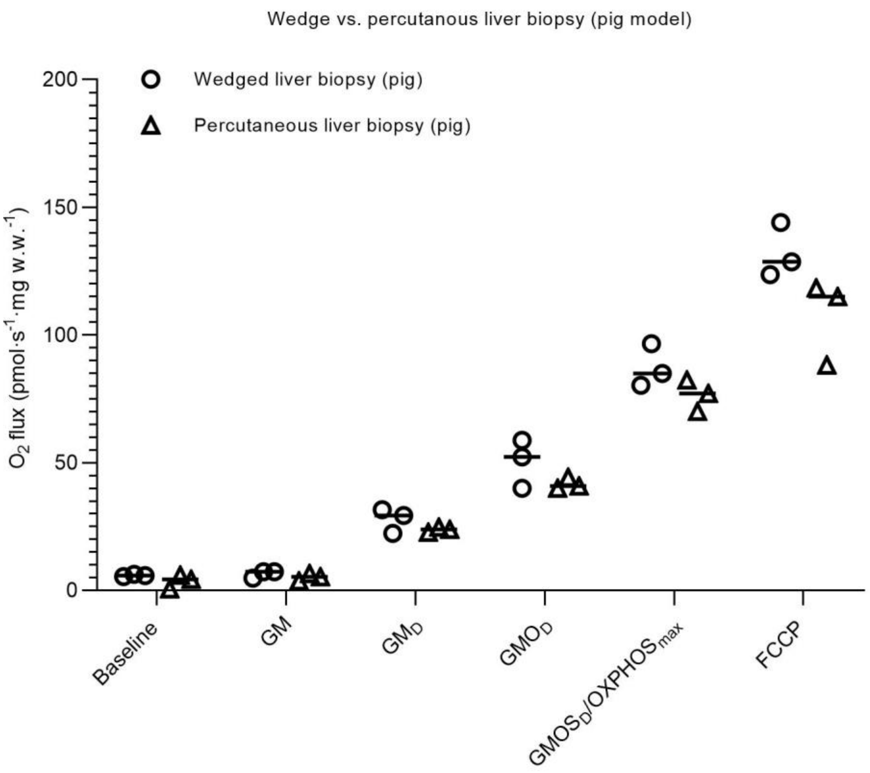

Comparison of mass specific O_2 fluxes in liver tissue obtained by different biopsy methods from one pig. Tissue was sampled pairwise and by mode of wedged liver biopsy (three biopsies) and percutaneous needle biopsy (three biopsies). All six biopsies were divided into two and studied in duplicates. The horizontal line represents the median.

Source data are provided as a Source Data file

Supplementary Figure S2: Flowchart

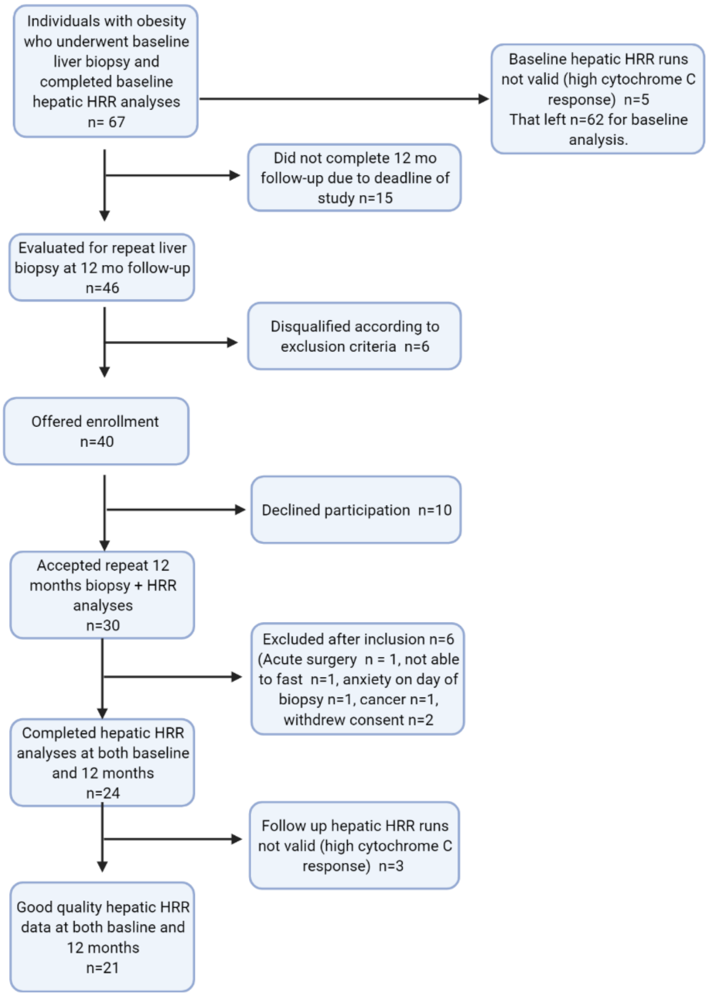

Supplement: Supplementary file 1 — Supplementary Information [file 41467_2022_30629_MOESM1_ESM.pdf]
