# Supplementary material for: Influence of NAFLD and bariatric surgery on hepatic and adipose tissue mitochondrial biogenesis and respiration
Source: Nat Commun. 2022 May 25;13:2931. doi: 10.1038/s41467-022-30629-5 (PMC9132900; doi:10.1038/s41467-022-30629-5)
Supplement: Supplementary file 3 — Supplementary Data 1 [file 41467_2022_30629_MOESM3_ESM.docx]

| **Supplementary Data S1.** Hepatic, visceral adipose tissue (VAT) and subcutaneous adipose tissue (SAT) respiratory rates (mass specific ,CS- and mtDNA/nDNA corrected) at baseline. | | | | |  |
| --- | --- | --- | --- | --- | --- |
|  | BASELINE | | | |  |
|  | **NAFL-** | **NAFL+** | **NASH** | **CON** |  |
| **Liver tissue SUIT P1**  **Mass specific respiratory rates**  **(pmol O_2_·s^-1^·mg. w.w.^-1^)** | n=30 | n=13 | n=13 | n=6 |  |
| **Baseline** | 3.5 (1.9-4.4) | 4.1 (2.3-5.4) | 3.8 (2.1-6.2) | 2.5 (1.8-3.7) |  |
| **Malate and Glutamate (GM)** | 5.1 (3.9-6.1) | 6.2 (5.0-7.8) | 4.3 (3.2-7.1) | 4.3 (3.0-6.3) |  |
| **ADP (GM_D_)** | 7.0 (5.3-8.9) | 9.0 (6.9-11.6) | 6.4 (4.1-9.7) | 7.6 (3.3-9.9) |  |
| **Octanoyl (GMO_D_)** | 12.6 (9.8-16.2) | 15.2 (11.3-19.9) | 11.5 (6.7-16.1) | 11.2 (7.3-16.6) |  |
| **Succinate (GMOS_D_/OXPHOS_max_)** | 31.1 (22.5-34.6) | 40.4 (26.3-47.2) | 31.7 (24.8-41.4) | 27.4 (18.6-38.8) |  |
| **FCCP** | 42.3 (32.8-54.0) | 55.6 (35.2-68.8) | 46.0 (30.8-65.2) | 35.8 (29.8-67.4) |  |
| **P/E (FCCP/ OXPHOS_max_)** | 0.67 (0.57-0.75) | 0.71 (0.56-0.78) | 0.73 (0.65-0.79) | 0.61 (0.56-0.73) |  |
| **RCR (GM_D_ /GM)** | 1.31 (1.11-1.65) | 1.36 (1.05-1.79) | 1.37 (1.21-1.62) | 1.48 (1.19-1.73) |  |
| **Liver tissue SUIT P1**  **mtDNA corrected respiratory rates**  **(pmol O_2_·s^-1^·mg. ^-1^·mtDNA/nDNA^-1^)** | n=30 | n=11 | n=12 | n=6 |  |
| **Baseline** | 0.007 (0.004-0.009) | 0.009 (0.006-0.013) | 0.008 (0.006-0.013) | 0.005 (0.003-0.009) |  |
| **Malate and Glutamate (GM)** | 0.011 (0.009-0.013) | 0.013 (0.011-0.016) | 0.011 (0.006-0.015) | 0.007 (0.005-0.014) ^§^ |  |
| **ADP (GM_D_)** | 0.015 (0.01-0.019) | 0.022 (0.015-0.027) | 0.015 (0.008-0.02) | 0.012 (0.007-0.021) |  |
| **Octanoyl (GMO_D_)** | 0.026 (0.018-0.034) | 0.034 (0.024-0.057) | 0.026 (0.014-0.034) | 0.023 (0.017-0.026) |  |
| **Succinate (GMOS_D_/OXPHOS_max_)** | 0.064 (0.048-0.075) | 0.079 (0.068-0.114) | 0.067 (0.051-0.085) | 0.051 (0.045-0.059) ^§^ |  |
| **FCCP** | 0.095 (0.076-0.118) | 0.135 (0.097-0.159) | 0.103 (0.072-0.142) | 0.079 (0.07-0.1) |  |
| **Liver tissue SUIT P1**  **CS corrected respiratory rates**  **(pmol O_2_·s^-1^·mg. ^-1^ ·CS activity^-1^)** | n=30 | n=13 | n=13 | n=6 |  |
| **Malate and Glutamate (GM)** | 0.48 (0.37-0.63) | 0.57 (0.44-0.91) | 0.43 (0.31-0.78) | 0.34 (0.24-0.65) |  |
| **ADP (GM_D_)** | 0.66 (0.45-0.84) | 0.86 (0.59-1.58) | 0.58 (0.43-1.09) | 0.60 (0.27-0.97) |  |
| **Octanoyl (GMO_D_)** | 1.24 (0.91-1.72) | 1.73 (0.80-2.30) | 1.01 (0.89-1.82) | 1.00 (0.63-1.54) |  |
| **Succinate (GMOS_D_/OXPHOS_max_)** | 2.98 (2.15-4.03) | 3.78 (2.20-5.10) | 3.87 (2.15-4.65) | 2.52 (1.65-3.17) |  |
| **FCCP** | 3.94 (3.32-6.67) | 6.08 (2.55-9.65) | 5.01 (2.78-6.11) | 3.39 (2.60-5.75) |  |
| **Liver tissue SUIT P2**  **Mass specific respiratory rates**  **(pmol O_2_·s^-1^·mg. w.w.^-1^)** | n=26 | n=16 | n=12 | n=7 |  |
| **Baseline** | 2.7 (1.9-4.7) | 5.0 (3.1-9.6) | 2.9 (1.6-5.3) | 2.2 (1.5-4.0) |  |
| **Malate and glutamate (GM)** | 4.3 (3.0-6.0) | 6.5 (4.4-10.9) | 4.5 (3.2-5.7) | 4.6 (2.7-5.8) |  |
| **ADP (GM_D_)** | 6.2 (4.3-7.5) | 7.0 (5.2-13.5) | 4.5 (4.0-5.8) | 6 (3.2-8.6) |  |
| **+Rotenone** | 2.9 (1.7-4.5) | 5.6 (2.8-11.3) | 2.6 (1.6-4.3) | 3.3 (2-4.8) |  |
| **+Succinate** | 24.0 (11.8-34.8) | 36.6 (26-42.3) | 26.5 (20.5-34.1) | 28.7 (18.1-41.2) |  |
| **+Antimycin A** | 3.8 (1.8-4.7) | 5.9 (3.5-8.9) | 2.3 (1.8-4.6) | 3.0 (2.0-5.6) |  |
| **+TMPD+asc** | 31.7 (27.5-38.9) | 26.2 (19.8-34.0) | 32.4 (13.5-49.1) | 37.1 (27.4-69.3) |  |
| **Liver tissue SUIT P2**  **mtDNA corrected respiratory rates**  **(pmolO_2_·s^-1^·mg.^-1^·mtDNA/nDNA^-1^)** | n=26 | n=14 | n=11 | n=7 |  |
| **Baseline** | 0,006 (0,004-0,011) | 0,012 (0,006-0,02) | 0,005 (0,003-0,008) | 0.004 (0.002-0.008) |  |
| **Malate and glutamate (GM)** | 0.010 (0.006-0.013) | 0.017 (0.01-0.023) | 0.008 (0.007-0.012) | 0.007 (0.006-0.016) |  |
| **ADP (GM_D_)** | 0.014 (0.009-0.017) | 0.019 (0.014-0.029) | 0.009 (0.008-0.013) ^§^ | 0.01 (0.007-0.022) |  |
| **+Rotenone** | 0.007 (0.003-0.01) | 0.015 (0.01-0.032) ^‡‡^ | 0.005 (0.003-0.008) ^§§^ | 0.005 (0.004-0.012) |  |
| **+Succinate** | 0.056 (0.025-0.074) | 0.076 (0.048-0.091) | 0.052 (0.043-0.079) | 0.052 (0.047-0.064) |  |
| **+Antimycin A** | 0.008 (0.004-0.012) | 0.013 (0.009-0.019) | 0.005 (0.004-0.008) ^§^ | 0.007 (0.003-0.015) |  |
| **+TMPD+asc** | 0.069 (0.057-0.081) | 0.063 (0.046-0.081) | 0.081 (0.039-0.097) | 0.069 (0.062-0.09) |  |
| **Liver tissue SUIT P2**  **CS corrected respiratory rates**  **(pmol O_2_·s^-1^·mg.^-1^ CS activity^-1^)** | n=25 | n=13 | n=12 | n=6 |  |
| **Malate and glutamate (GM)** | 0.41 (0.28-0.58) | 0.81 (0.34-1.20) | 0.40 (0.34-0.54) | 0.35 (0.26-0.78) |  |
| **ADP (GM_D_)** | 0.56 (0.38-0.79) | 0.95 (0.39-1.33) | 0.48 (0.38-0.58) | 0.56 (0.33-1.10) |  |
| **+Rotenone** | 0.28 (0.15-0.44) | 0.62 (0.26-1.06) | 0.26 (0.18-0.39) | 0.26 (0.20-0.60) |  |
| **+Succinate** | 2.51 (1.15-3.62) | 3.33 (2.36-4.80) | 2.53-1.87-3.52) | 2.65 (1.90-3.22) |  |
| **+Antimycin A** | 0.36 (0.17-0.46) | 0.56 (0.29-0.83) | 0.22 (0.14-0.42) | 0.22 (0.12-0.75 |  |
| **+TMPD+asc** | 3.02 (2.56-3.68) | 1.76 (1.31-2.96) | 2.77 (2.23-2.75) | 3.55 (2.37-4.92) |  |
| **Visceral adipose tissue SUIT P1**  **Mass specific respiratory rates**  **(pmol O_2_·s^-1^·mg. w.w.^-1^)** | n=30 | n=12 | n=14 | n=9 |  |
| **Baseline** | 0.02 (0-0.03) | 0.03 (0-0.06) | 0 (0-0.02) | 0.02 (0.01-0.04) |  |
| **Malate and Glutamate (GM)** | 0.06 (0.05-0.07) | 0.05 (0.02-0.08) | 0.05 (0.03-0.07) | 0.1 (0.06-0.15) |  |
| **ADP (GM_D_)** | 0.4 (0.27-0.49) | 0.31 (0.14-0.5) | 0.28 (0.14-0.46) | 0.56 (0.43-0.73) ^†^ |  |
| **Octanoyl (GMO_D_)** | 0.67 (0.49-0.94) | 0.55 (0.25-0.83) | 0.48 (0.29-0.75) | 1.13 (0.76-1.35) ^§,†^ |  |
| **Succinate (GMOS_D_/OXPHOS_max_)** | 1.37 (0.82-1.72) | 1.12 (0.73-1.63) | 1.11 (0.63-1.57) | 1.86 (1.47-2.91) ^§,†^ |  |
| **FCCP** | 1.52 (0.99-1.98) | 1.29 (0.89-1.82) | 1.39 (0.78-1.83) | 2.71 (1.74-3.45) ^§,†^ |  |
| **P/E (FCCP/ OXPHOS_max_)** | 0.84 (0.8-0.87) | 0.86 (0.81-0.91) | 0.83 (0.8-0.88) | 0.79 (0.75-0.86) |  |
| **RCR (GM_D_ /GM)** | 6.0 (5.02-8.50) | 5.50 (4.43-7.75) | 5.84 (2.40-7.40) | 6.0 (4.81-8.30) |  |
| **Visceral adipose tissue SUIT P1**  **mtDNA corrected respiratory rates**  **(pmol O_2_·s^-1^·mg.^-1^·mtDNA/nDNA^-1^)** | n=29 | n=10 | n=14 | n=8 |  |
| **Baseline** | 0.0001 (0-0.0001) | 0.0001 (0-0.0002) | 0 (0-0.0001) | 0.0001 (0-0.0001) |  |
| **Malate and Glutamate (GM)** | 0.0002  (0.0002-0.0003) | 0.0002  (0.0001-0.0003) | 0.0002  (0.0001-0.0003) | 0.0003  (0.0002-0.0005) |  |
| **ADP (GM_D_)** | 0.0015  (0.001-0.0019) | 0.0011  (0.0007-0.0017) | 0.0015  (0.0005-0.0019) | 0.0018  (0.0011-0.003) |  |
| **Octanoyl (GMO_D_)** | 0.0026  (0.0017-0.0037) | 0.0019  (0.0012-0.003) | 0.0025  (0.001-0.0033) | 0.0037  (0.0022-0.0059) |  |
| **Succinate (GMOS_D_/OXPHOS_max_)** | 0.0049  (0.0029-0.0063) | 0.0046  (0.0032-0.0059) | 0.0047  (0.0021-0.0067) | 0.0074  (0.0046-0.01) |  |
| **FCCP** | 0.0059  (0.0035-0.0074) | 0.0051  (0.0038-0.0068) | 0.0068  (0.0026-0.0079) | 0.0093  (0.0058-0.0123) |  |
| **Subcutaneous adipose tissue SUIT P1**  **Mass specific respiratory rates**  **(pmol O_2_·s^-1^·mg. w.w.^-1^)** | n=29 | n=13 | n=14 | n=9 |  |
| **Baseline** | 0.01 (0-0.03) | 0.02 (0.01-0.05) | 0 (0-0.01) | 0.01 (0-0.04) |  |
| **Malate and Glutamate (GM)** | 0.04 (0.03-0.07) | 0.05 (0.03-0.07) | 0.05 (0.03-0.06) | 0.09 (0.06-0.12) ^‡,†^ |  |
| **ADP (GM_D_)** | 0.30 (0.26-0.38) | 0.30 (0.18-0.41) | 0.29 (0.21-0.35) | 0.52 (0.49-0.76) ^‡‡‡ §§§,†††^ |  |
| **Octanoyl (GMO_D_)** | 0.43 (0.35-0.54) | 0.44 (0.22-0.54) | 0.42 (0.33-0.51) | 0.74 (0.66-0.82) ^‡‡‡,§§,†††^ |  |
| **Succinate (GMOS_D_/OXPHOS_max_)** | 0.87 (0.68-1.03) | 0.87 (0.65-0.93) | 0.81 (0.73-1) | 1.33 (1.29-2.21) ^‡‡‡,§§§,†††^ |  |
| **FCCP** | 1.08 (0.86-1.36) | 1.07 (0.83-1.13) | 1.03 (0.92-1.29) | 1.69 (1.53-2.78) ^‡‡‡,§§§,†††^ |  |
| **P/E (FCCP/ OXPHOS_max_)** | 0.78 (0.75-0.84) | 0.83 (0.76-0.86) | 0.77 (0.77-0.83) | 0.82 (0.77-0.84) |  |
| **RCR (GM_D_ /GM)** | 5.40 (4.25-8.32) | 6.71 (3.71-10.00) | 6.00 (4.08-11.17) | 7.00 (5.52-9.15) |  |
| **Subcutaneous adipose tissue (SUIT P1)**  **mtDNA corrected respiratory rates**  **(pmol O_2_·s^-1^·mg.^-1^·mtDNA/nDNA^-1^)** | n=27 | n=13 | n=13 | n=9 |  |
| **Baseline** | 0.0001 (0-0.0001) | 0.0001 (0-0.0002) | 0 (0-0.0001) | 0 (0-0.0001) |  |
| **Malate and Glutamate (GM)** | 0.0002  (0.0001-0.0003) | 0.0002  (0.0001-0.0002) | 0.0002  (0.0001-0.0003) | 0.0003  (0.0002-0.0005) ^‡‡,§§,†^ |  |
| **ADP (GM_D_)** | 0.0014  (0.001-0.0016) | 0.0011  (0.0007-0.0016) | 0.0012  (0.0009-0.0017) | 0.0021  (0.0018-0.0032) ^‡,§§,†^ |  |
| **Octanoyl (GMO_D_)** | 0.0018  (0.0014-0.0025) | 0.0016  (0.0009-0.0023) | 0.0018  (0.0014-0.0025) | 0.0029  (0.0024-0.0031) ^‡‡,§§,††^ |  |
| **Succinate (GMOS_D_/OXPHOS_max_)** | 0.0036  (0.003-0.0046) | 0.0032  (0.0025-0.0044) | 0.0035  (0.0026-0.0047) | 0.0053  (0.0048-0.0088) ^‡,§§,†^ |  |
| **FCCP** | 0.0047  (0.0038-0.0058) | 0.0038  (0.0032-0.0057) | 0.0047  (0.0033-0.0059) | 0.0069  (0.0058-0.0111)^‡,§§,†^ |  |
| Data are presented as medians (IQR). P-values (2-sided) are Kruskal-Wallis with correction for multiple comparison.  SUIT P1; substrate-inhibitor protocol 1, SUIT P2; substrate-inhibitor protocol 2; w.w., wet weight; ADP, adenosine diphosphate; OXPHOS_max_, maximal mitochondrial oxidative phosphorylation; FCCP, *p*-triflouromethoxyphenylhydrazone; RCR, respiratory control ratio; mtDNA, mitochondrial deoxyribonucleic acid; nDNA, nuclear DNA; TMPD, *N*,*N*,*N*,*N’* -tetramethyl-*p*-phenylenediamine; asc, ascorbate.  ‡, ‡‡, ‡‡‡ denotes statistical significance (P <0.05, 0.01, 0.001 respectively) compared with NAFL-.  §, §§, §§§ denotes statistical significance (P <0.05, 0.01, 0.001 respectively) compared with NAFL+  †, ††, ††† denotes statistical significance (P <0.05, 0.01, 0.001 respectively) compared with NASH | | | | | |
