# Supplementary material for: Influence of NAFLD and bariatric surgery on hepatic and adipose tissue mitochondrial biogenesis and respiration
Source: Nat Commun. 2022 May 25;13:2931. doi: 10.1038/s41467-022-30629-5 (PMC9132900; doi:10.1038/s41467-022-30629-5)
Supplement: Supplementary file 4 — Supplementary Data 2 [file 41467_2022_30629_MOESM4_ESM.docx]

| **Supplementary Data S2.** Hepatic mass specific and mtDNA corrected respiratory fluxes at  baseline vs. follow-up 12 months after bariatric surgery in 21 OBE study subjects. | | |  |
| --- | --- | --- | --- |
| **Liver tissue SUIT P1**  **Mass specific flux**  **(pmolO_2_·s^-1^·mg·w.w. ^-1^)** | **OBE baseline**  **(n=21)** | **OBE follow-up**  **(n=21)** | **P-value** |
| **Baseline** | 3.7 (2.3-4.5) | 1.3 (0.4-3.6) | 0.073 |
| **Malate and Glutamate (GM)** | 5.5 (4.7-6.2) | 4.7 (2.9-6.2) | 0.375 |
| **ADP (GM_D_)** | 8.5 (5.5-10.2) | 7.8 (6.0-9.4) | 0.455 |
| **Octanoyl (GMO_D_)** | 13.2 (10.6-16.3 | 14.7 (11.6-16.3) | 0.931 |
| **Succinate (GMOS_D_/OXPHOS_max_)** | 31.6 (24.4-37.1) | 39.7 (28.8-53.4) | 0.099 |
| **FCCP** | 44.9 (31.7-54.0) | 65.8 (57.3-73.0) | *P* < 0.01 |
| **P/E (FCCP/ OXPHOS_max_)** | 0.69 (0.61-0.76) | 0.57 (0.50-0.59) | *P* < 0.05 |
| **RCR (GM_D_/GM)** | 1.55 (1.17-1.89) | 1.65 (1.21-1.88) | *P*= 0.958 |
| **mtDNA/nDNA count, average** | 481 (441 ─527) | 660 (431─753) | P = 0.012 |
| **Liver tissue SUIT P1**  **mtDNA corrected respiratory rates**  **(pmol O_2_·s^-1^·mg. ^-1^·mtDNA/nDNA^-1^)** | n=21 | n=21 |  |
| **Malate and Glutamate (GM)** | 0.012 (0.011-0.013) | 0.007 (0.005-0.013) | 0.107 |
| **ADP (GM_D_)** | 0.018 (0.013-0.022) | 0.013 (0.009-0.016) | 0.099 |
| **Octanoyl (GMO_D_)** | 0.030 (0.023-0.034) | 0.025 (0.019-0.034) | 0.198 |
| **Succinate (GMOS_D_/OXPHOS_max_)** | 0.066 (0.052-0.094) | 0.059 (0.045-0.089) | 0.212 |
| **FCCP** | 0.095 (0.076-0.126) | 0.096 (0.080-0.148) | 0.088 |
| **Liver tissue SUIT P2**  **Mass specific flux**  **(pmolO_2_·s^-1^·mg·w.w. ^-1^)** | n=16 | n=19 |  |
| **Baseline** | 3.1 (1.9-7.4) | 2.5 (0.2-3.5) | 0.033 |
| **Malate and glutamate (GM)** | 4.9 (2.6-9.7) | 4.5 (3.4-5.8) | 0.300 |
| **ADP (GM_D_)** | 5.6 (3.6-7.8) | 8.0 (5.1-9.8) | 0.198 |
| **+Rotenone** | 2.9 (2.1-5.8) | 2.8 (2.2-4.0) | 0.087 |
| **+Succinate** | 22.4 (11.3-29.2) | 35.7 (27.9-44.9) | *P* < 0.05 |
| **+Antimycin A** | 3.3 (1.9-5.2) | 4.1 (2.1-5.6) | 1.0 |
| **+TMPD+asc** | 22.4 (19.1-30.6) | 50.7 (40.7-55.8) | *P* < 0.05 |
| **Liver tissue SUIT P2**  **mtDNA corrected respiratory rates**  **(pmol O_2_·s^-1^·mg. ^-1^·mtDNA/nDNA^-1^)** | n=15 | n=19 |  |
| **Malate and glutamate (GM)** | 0.012 (0.005-0.022) | 0.008 (0.005-0.012) | 0.117 |
| **ADP (GM_D_)** | 0.136 (0.006-0.019) | 0.013 (0.008-0.022) | 0.583 |
| **+Rotenone** | 0.009 (0.004-0.015) | 0.004 (0.004-0.010) | 0.050 |
| **+Succinate** | 0.044 (0.021-0.057) | 0.057 (0.044-0.095= | 0.131 |
| **+Antimycin A** | 0.008 (0.003-0.013) | 0.006 (0.003-0.009) | 0.799 |
| **+TMPD+asc** | 0.057 (0.055-0.061) | 0.079 (0.058-0.092) | < 0.05 |

Data are presented as medians (IQR). P-values (2-sided) are Wilcoxon signed rank test.

ADP, adenosine diphosphate; VAT, visceral adipose tissue; SAT, subcutaneous adipose tissue; SUIT P1, substrate-inhibitor protocol 1; w.w., wet weight; ADP, adenosine diphosphate; OXPHOS, mitochondrial oxidative phosphorylation; FCCP, *p*-triflouromethoxyphenylhydrazone; RCR, respiratory control ratio; SUIT P2, substrate-inhibitor protocol 2, TMPD, *N*,*N*,*N’*,*N’* -tetramethyl-*p*-phenylenediamine; asc, ascorbate
