# Supplementary material for: Influence of NAFLD and bariatric surgery on hepatic and adipose tissue mitochondrial biogenesis and respiration
Source: Nat Commun. 2022 May 25;13:2931. doi: 10.1038/s41467-022-30629-5 (PMC9132900; doi:10.1038/s41467-022-30629-5)
Supplement: Supplementary file 5 — Supplementary Data 3 [file 41467_2022_30629_MOESM5_ESM.docx]

**Supplementary Data S3**. Hepatic, visceral adipose tissue (VAT) and subcutaneous adipose tissue (SAT) respiratory rates (mass specific, CS- and mtDNA/nDNA corrected) in study subjects with obesity with or without diabetes type 2 at baseline.

|  | **Diabetes type 2 at**  **baseline= no** | **Diabetes type 2 at baseline =yes** | **P-value** |
| --- | --- | --- | --- |
| **Liver tissue SUIT P1**  **Mass specific respiratory rates**  **(pmol O_2_·s^-1^·mg. w.w.^-1^)** | n=43 | n=13 |  |
| Malate and Glutamate (GM) | 5.3 (4.1-6.8) | 5.3 (3.5-6.2) | 0.426 |
| ADP (GM_D_) | 7.5 (5.4-9.2) | 8.0 (4.3-10.3) | 0.915 |
| Octanoyl (GMO_D_) | 13.0 (9.9-17.2) | 12.0 (7.1-15.9) | 0.313 |
| Succinate (GMOS_D_/OXPHOS_max_) | 31.8 (25.1-37.5) | 25.2 (22.6-42.1) | 0.900 |
| FCCP | 44.9 (38.6-63.0) | 49.0 (27.3-70.9) | 0.559 |
| **Liver tissue SUIT P1**  **mtDNA corrected respiratory rates (pmol O_2_·s^-1^·mg.^-1^·mtDNA/nDNA^-1^)** | n=42 | n=11 |  |
| Malate and Glutamate (GM) | 0.011 (0.009-0.014) | 0.012 (0.009-0,014) | 0.776 |
| ADP (GM_D_) | 0.016 (0.010-0.020) | 0.017 (0.011-0.024) | 0.554 |
| Octanoyl (GMO_D_) | 0.029 (0.021-0.035) | 0.024 (0.018-0.035) | 0.667 |
| Succinate (GMOS_D_/OXPHOS_max_) | 0.067 (0.053-0.083) | 0.076 (0.048-0.095) | 0.759 |
| FCCP | 0.097 (0.080-0.138) | 0.102 (0.069-0.125) | 0.858 |
| **Liver tissue SUIT P2**  **Mass specific respiratory rates**  **(pmol O_2_·s^-1^·mg. w.w.^-1^)** | n=40 | n=14 |  |
| Malate and glutamate (GM) | 4.5 (3.3-6.3) | 5.0 (3.5-7.5) | 0.547 |
| ADP (GM_D_) | 6.2 (4.1-8.1) | 5.8 (4.8-8.6) | 0.477 |
| +Rotenone | 3.3 (2.0-5.2) | 6.2 (4.1-8.1) | 0.896 |
| +Succinate | 26.7 (19.1-35.5) | 30.3 (20.3-38.4) | 0.489 |
| +Antimycin A | 3.9 (2.3-4.8) | 3.6 (2.1-6.8) | 0.901 |
| +TMPD+asc | 30.9 (24.9-36.0) | 32.4 (19.0-49.1) | 0.784 |
| **Liver tissue SUIT P2**  **mtDNA corrected respiratory rates**  **(pmolO_2_·s^-1^·mg.^-1^·mtDNA/nDNA^-1^)** | n=39 | n=12 |  |
| Malate and glutamate (GM) | 0.011 (0.007-0.015) | 0.014 (0.007-0019) | 0.756 |
| ADP (GM_D_) | 0.014 (0.008-0.018) | 0.014 (0.010-0.023 | 0.505 |
| +Rotenone | 0.007 (0.003-0.011) | 0.008 (0.006-0.013) | 0.650 |
| +Succinate | 0.062 (0.039-0.077) | 0.064 (0.043-0.101) | 0.352 |
| +Antimycin A | 0.008 (0.004-0.012) | 0.008 (0.005-0.012) | 0.907 |
| +TMPD+asc | 0.068 (0.057-0.082) | 0.081 (0.060-0.102) | 0.216 |
| **Visceral adipose tissue SUIT P1**  **Mass specific respiratory rates**  **(pmol O_2_·s^-1^·mg. w.w.^-1^)** | n=43 | n=13 |  |
| Malate and Glutamate (GM) | 0.05(0.03-0.07) | 0.05 (0.03-0.08) | 0.899 |
| ADP (GM_D_) | 0.40 (0.26-0.50) | 0.29 (0.14-0.46) | 0.308 |
| Octanoyl (GMO_D_) | 0.67 (0.45-0.87) | 0.62 (0.21-0.70) | 0.227 |
| Succinate (GMOS_D_/OXPHOS_max_) | 1.21 (0.82-1.69) | 1.12 (0.57-1.44) | 0.225 |
| FCCP | 1.50 (0.99-1.96) | 1.36 (0.71-1.65) | 0.213 |
| **Visceral adipose tissue SUIT P1**  **mtDNA corrected respiratory rates (pmol O_2_·s^-1^·mg.^-1^·mtDNA/nDNA^-1^)** | n=42 | n=11 |  |
| Malate and Glutamate (GM) | 0.0002 (0.0001-0.0003) | 0.0002 (0.0001-0.0003) | 0.930 |
| ADP (GM_D_) | 0.0015 (0.0009-0.0019) | 0.0020 (0.0005-0.0018) | 0.456 |
| Octanoyl (GMO_D_) | 0.0025 (0.0016-0.0032) | 0.0024 (0.0009-0.0032) | 0.671 |
| Succinate (GMOS_D_/OXPHOS_max_) | 0.0049 (0.0030-0.0060) | 0.0047 (0.0019-0.0064) | 0.803 |
| FCCP | 0.0057 (0.0037-0.0074) | 0.0055 (0.0023-0.0077) | 0.617 |
| **Subcutaneous adipose tissue SUIT P1**  **Mass specific respiratory rates**  **(pmol O_2_·s^-1^·mg. w.w.^-1^)** | n=43 | n=13 |  |
| Malate and Glutamate (GM) | 0.04 (0.03-0.07) | 0.05 (0.02-0.07) | 0.750 |
| ADP (GM_D_) | 0.30 (0.26-0.38) | 0.30 (0.18-0.38) | 0.607 |
| Octanoyl (GMO_D_) | 0.44 (0.35-0.54) | 0.42 (0.22-0.54) | 0.565 |
| Succinate (GMOS_D_/OXPHOS_max_) | 0.85 (0.69-0.95) | 0.87 (0.64-1.02) | 0.797 |
| FCCP | 1.08 (0.95-1.17) | 1.01 (0.81-1.34) | 0.934 |
| **Subcutaneous adipose tissue SUIT P1**  **mtDNA corrected respiratory rates (pmol O_2_·s^-1^·mg.^-1^·mtDNA/nDNA^-1^)** | n=41 | n=12 |  |
| Malate and Glutamate (GM) | 0.002 (0.0001-0.0002) | 0.0002 (0.0001-0.0003) | 0.702 |
| ADP (GM_D_) | 0.0014 (0.0010-0.0016) | 0.0014 (0.0009-0.0016) | 0.625 |
| Octanoyl (GMO_D_) | 0.0017 (0.0014-0.0024) | 0.0017 (0.0014-0.0024) | 0.529 |
| Succinate (GMOS_D_/OXPHOS_max_) | 0.0035 (0.0029-0.0046) | 0.0034 (0.0024-0.0048) | 0.761 |
| FCCP | 0.0045 (0.0036-0.0058) | 0.0045 (0.0031-0.0064) | 0.820 |

Data are presented as medians (IQR). P-values (2-sided) are Mann-Whitney U test

SUIT P1; substrate-inhibitor protocol 1, SUIT P2; substrate-inhibitor protocol 2; w.w., wet weight; ADP, adenosine diphosphate; OXPHOS_max_, maximal mitochondrial oxidative phosphorylation; FCCP, *p*-triflouromethoxyphenylhydrazone; RCR, respiratory control ratio; mtDNA, mitochondrial deoxyribonucleic acid; nDNA, nuclear DNA; TMPD, *N*,*N*,*N*,*N’* -tetramethyl-*p*-phenylenediamine; asc, ascorbate.
