# Supplementary material for: Influence of NAFLD and bariatric surgery on hepatic and adipose tissue mitochondrial biogenesis and respiration
Source: Nat Commun. 2022 May 25;13:2931. doi: 10.1038/s41467-022-30629-5 (PMC9132900; doi:10.1038/s41467-022-30629-5)
Supplement: Supplementary file 6 — Reporting Summary [file 41467_2022_30629_MOESM6_ESM.pdf]

Corresponding author(s): Julie Steen Pedersen

Last updated by author(s): April 4th, 2022

Reporting Summary

Nature Portfolio wishes to improve the reproducibility of the work that we publish. This form provides structure for consistency and transparency in reporting. For further information on Nature Portfolio policies, see our Editorial Policies and the Editorial Policy Checklist.

Statistics

For all statistical analyses, confirm that the following items are present in the figure legend, table legend, main text, or Methods section.

n/a	Confirmed	
☐	☒	The exact sample size (*n*) for each experimental group/condition, given as a discrete number and unit of measurement
☐	☒	A statement on whether measurements were taken from distinct samples or whether the same sample was measured repeatedly
☐	☒	The statistical test(s) used AND whether they are one- or two-sided<br>*Only common tests should be described solely by name; describe more complex techniques in the Methods section.*
☐	☒	A description of all covariates tested
☐	☒	A description of any assumptions or corrections, such as tests of normality and adjustment for multiple comparisons
☐	☒	A full description of the statistical parameters including central tendency (e.g. means) or other basic estimates (e.g. regression coefficient) AND variation (e.g. standard deviation) or associated estimates of uncertainty (e.g. confidence intervals)
☐	☒	For null hypothesis testing, the test statistic (e.g. *F*, *t*, *r*) with confidence intervals, effect sizes, degrees of freedom and *P* value noted<br>*Give P values as exact values whenever suitable.*
☒	☐	For Bayesian analysis, information on the choice of priors and Markov chain Monte Carlo settings
☒	☐	For hierarchical and complex designs, identification of the appropriate level for tests and full reporting of outcomes
☒	☐	Estimates of effect sizes (e.g. Cohen's *d*, Pearson's *r*), indicating how they were calculated

Our web collection on statistics for biologists contains articles on many of the points above.

Software and code

Policy information about availability of computer code

Data collection	Oroboros Instruments (Innsbruck, Austria) Software: Oroboros DatLab 6.0
Data analysis	NDP.view2 ((Hamamatsu, Hamamatsu, Japan)<br>IBM SPSS Statistics 25 64-bit.

For manuscripts utilizing custom algorithms or software that are central to the research but not yet described in published literature, software must be made available to editors and reviewers. We strongly encourage code deposition in a community repository (e.g. GitHub). See the Nature Portfolio guidelines for submitting code & software for further information.

Data

Policy information about availability of data

All manuscripts must include a data availability statement. This statement should provide the following information, where applicable:

- Accession codes, unique identifiers, or web links for publicly available datasets
- A description of any restrictions on data availability
- For clinical datasets or third party data, please ensure that the statement adheres to our policy

Source Data to figures are provided with this paper. Other data that support the findings of this study are available upon reasonable request from the corresponding author [Julie Steen Pedersen, contact information: julie.steen.pedersen@regionh.dk] on the condition that individual approval can be provided by

the Danish Data Protection Agency. The timeframe for response to requests is dependent on the case processing time at the Danish Data Protection Agency. Some data are not publicly available due to restrictions set by the Danish Data Protection Agency as public sharing and sharing of large sets of metadata could compromise research participant privacy/consent and hence violate our Data Agreement with the Danish Data Protection Agency.

Field-specific reporting

Please select the one below that is the best fit for your research. If you are not sure, read the appropriate sections before making your selection.

☒ Life sciences ☐ Behavioural & social sciences ☐ Ecological, evolutionary & environmental sciences

For a reference copy of the document with all sections, see nature.com/documents/nr-reporting-summary-flat.pdf

Life sciences study design

All studies must disclose on these points even when the disclosure is negative.

Sample size	Sample size was determined by use of R by which we dteromined af sample size of 60 with n=15 per group (alpha 0.050, power 0.80). We ended up with more than n=15 in some groups and n>15 in other groups. However, it is still the largest human dataset on ex vivo respiration in liver and adipose tissue
Data exclusions	We included more participants than what we present data from in this paper. We made thourough data quality check on all respiratory data and excluded respiratory data (and hence participants) had they e.g. a too high cytochrome C response which indicated compromised tissue quality. Also, we had a power break down during 2 HRR analyses (same day) and when the oxygraphs were up and running again shortly after we were unsure of the measurements and excluded these two participants as well.<br>Initially we included 11 control study subjects but two subjects had mild steatosis in their liver biopsy and we excluded these subjects and their HRR measurements.
Replication	All HRR analyses were performed in duplicates at baseline (singlicates 12 months after surgery) and mtDNA analyses were performed in triplicates.<br>We replicated the data from wedge vs percutaneous liver biopsy in a pig model to ensure that the increase in respiration observed 12 months after surgery was not due to difference in sampling methods.
Randomization	Participants were divided into groups based on their NAFLD severity status (evaluated in teir baseline liver biopsy)
Blinding	The lab technicians who performed the HRR measurements were completely blinded to patient details.<br>HRR datacuration was performed by a person blinded to all patient detail.<br>The PI was unaware of NAFLD status in the patients at the time of HRR analyses.

Reporting for specific materials, systems and methods

We require information from authors about some types of materials, experimental systems and methods used in many studies. Here, indicate whether each material, system or method listed is relevant to your study. If you are not sure if a list item applies to your research, read the appropriate section before selecting a response.

Materials & experimental systems

n/a	Involved in the study	
☐	☒	Antibodies
☒	☐	Eukaryotic cell lines
☒	☐	Palaeontology and archaeology
☐	☒	Animals and other organisms
☐	☒	Human research participants
☒	☐	Clinical data
☒	☐	Dual use research of concern

Methods

n/a	Involved in the study	
☒	☐	ChIP-seq
☒	☐	Flow cytometry
☒	☐	MRI-based neuroimaging

Antibodies

Antibodies used	cytoplasmatic pan-macrophage marker CD68 (Ready-to-use monoclonal mouse antibody, clone KP1, DAKO, Glostrup, Denmark)
Validation	The CD68 antibody is a ready to use (RTU) antibody, and the staining performed according to the vendor's (Agilent's) instructions. Validation statements for the primary antibody is available on Agilent's homepage (https://www.agilent.com/en/product/immunohistochemistry/antibodies-controls/primary-antibodies/cd68-(dako-omnis)-76223) and the performance of the antibody with optimized protocols are available on the international immunohistochemistry quality assessment scheme NordiQC's website https://www.nordiqc.org/epitope.php?id=33

Animals and other organisms

Policy information about studies involving animals; ARRIVE guidelines recommended for reporting animal research

Laboratory animals	One female Danish Landrace pig, age 18 weeks, weight 51 kg.
Wild animals	The study did not involve wild animals
Field-collected samples	Samples were not collected from the field.
Ethics oversight	All necessary approvals had been obtained (license number 2018-15-0201-01608). All animal experiments were performed in accordance with the Danish law for the protection of animals and the investigation conformed to the guidelines from Directive 2010/63/EU of the European Parliament on the protection of laboratory animals and to the ARRIVE-guidelines. The study had been reviewed and approved by the regional Animal Welfare Inspectorate in Denmark.

Note that full information on the approval of the study protocol must also be provided in the manuscript.

Human research participants

Policy information about studies involving human research participants

Population characteristics	The study cohort comprised of 62 indivduals with obesity (41 females, 21 males) median age 44 years (IQR 39-57), who underwent primary bariatric surgery (either Roux-en-Y Gastric Bypass (n=29) or Sleeve Gastrectomy (n=33) at Copenhagen University Hospital Hvidovre 2016-2019. Study subjects were stratified into groups based on their NAFLD status at the study specific baseline liver biopsy sampled during the bariatric procedure. Visceral- and subcutaneous adipose tissue were also collected during surgery.<br>In addition we included 9 healthy, normal weight participants (7 females, 2 males), median age 39 years (IQR 24-43) who underwent planned removal of their gall bladder and sampled liver- and adipose tissue during their surgery.
Recruitment	All study subjects (including controls) were enrolled between December 2016 and September 2019 at Copenhagen University Hospital Hvidovre. We enrolled consecutively from the surgical out-patient clinic at the Gastro Unit 1-2 weeks prior to their bariatric surgery. Here they attended the last infomation meeting prior to their surgery. The PI of the study was given 3 minutes to introduce the study in plenum. Afterwards the PI sat in a room next to the meeting room. Subsequently, potential study subjects could seek out the PI and receive oral and written information. All study subjects were given a minimum of 24 hours of consideration.<br><br>The individuals with obesity fulfilled the existing criteria for bariatric surgery issued by the Danish Health Authorities. The PI had no influence of mode of surgery (gastric bypass vs sleeve gastrectomy) which had already been decided by the expert endocrinologist. At the time of enrollment the PI was blinded to details of the surgical procedure and details (eg comorbidity) of the potential study subject. Only if a potential study subject expressed interest in participation details of e.g. comorbidity would be disclosed to the PI (also in order to screen for exlusion criteria). Due to the consecutive mode of enrollment in combination with blinding to details of the potential study subject we believe that the introduction of selection bias to be minimal.
Ethics oversight	Ethical Committee Capital Region Denmark (approvals H-16030784 and H-16030782) and the Danish Data Protection Agency (approvals P-2019-514 and P-2020-606)

Note that full information on the approval of the study protocol must also be provided in the manuscript.
